# Biodegradation of screenings from sewage treatment by white rot fungi

**DOI:** 10.1186/s40694-025-00198-5

**Published:** 2025-05-14

**Authors:** Anna Civzele, Alise Anna Stipniece-Jekimova, Linda Mezule

**Affiliations:** Water Systems and Biotechnology Institute, Riga Technical University, Riga, Latvia

**Keywords:** Lignocellulolytic enzymes, Screenings, Waste treatment, Wastewater treatment, White rot fungi

## Abstract

Wastewater treatment plants (WWTPs), particularly activated sludge systems, generate significant amounts of various types of waste, including screenings, primary sludge, and secondary sludge. While substantial research has been conducted on the recovery and valorization of sewage sludge, the treatment and utilization of screenings remain underexplored. In response, this study investigates the potential of white rot fungi to degrade cellulose-containing waste screened during the preliminary treatment and examines the production patterns of lignocellulolytic enzymes in the presence of this waste. The studied fungi exhibited variable enzymatic responses depending on the type of substrate, however, their adaptability highlighted the potential in fungal-mediated bioconversion processes. *P. dryinus* and *T. versicolor* were identified as strong and adaptive candidates for oxidative enzyme production, with *P. dryinus* showing laccase activity up to 1691.75 ± 12.22 U/mg and degrading 44.46% of carbohydrates in tested screenings. *I. lacteus* and *B. adusta* were predominantly observed in cellulolytic enzyme production, with *B. adusta* ensuring a 43.49% reduction in carbohydrate content of screenings. As a result of fungal cultivation in WWTP waste, the production potential of 34 to 46 kg of sugars per ton of screenings was determined. Therefore, the study presents a promising approach for the sustainable treatment of screenings and the development of waste management and resource recovery strategies for WWTP-derived waste.

**Supplementary Information:**

The online version contains supplementary material available at 10.1186/s40694-025-00198-5.

## Introduction

Wastewater treatment is a critical process for protecting environmental and public health, as it removes harmful contaminants, pathogens, and pollutants from the water before its release into the environment or reuse (Silva 2023). The activated sludge process is the most widely used wastewater treatment technology, employed worldwide for more than a century (Yang et al. 2020). However, despite the numerous advantages of activated sludge systems, these WWTPs generate significant amounts of waste (Krishna et al. 2022; Valchev et al. 2024), which presents considerable challenges in terms of disposal and management.

Throughout the stages of the wastewater treatment plant (WWTP), diverse types of waste with variable compositions are generated. During preliminary treatment, waste containing large debris such as sanitation textiles, toilet paper, organic material like leftovers, as well as plastics, grit, and other solid materials (Gregor et al. 2013), is removed from the influent wastewater through coarse screens. In the primary treatment stage, primary sludge is produced, which is composed of suspended solids derived from primary settlement tanks. This sludge is high in organic content (Lin et al. 2018), making it a potential source for energy recovery (Capodaglio and Callegari 2023). Then, the secondary treatment process generates the secondary sludge, or waste activated sludge, predominantly containing excess microbial biomass produced during the biological treatment process (Baroutian et al. 2013).

As the global population and urbanization grow (Ritchie and Rodés-Guirao 2024), the increase in global water demand will unavoidably lead to a corresponding rise in wastewater production, which is expected to increase by 24% by 2030 and by 51% by 2050 (Qadir et al. 2020; Ryder 2017). This rise in wastewater production creates additional pressure on existing treatment facilities, intensifying the challenge of managing and disposing waste generated during the wastewater treatment process. Fortunately, sewage sludge due to the promising composition and compatibility with anaerobic digestion has resulted in extensive interest to use this waste for resource recovery and energy production (Gao et al. 2020; Bagheri et al. 2023; Enebe et al. 2023). However, wastewater screenings are the waste produced by WWTPs that do not yet have a valorization pathway and are generally ending up in landfills (De la Torre-Bayo et al. 2022). Consequently, this can pose a hazard to ecosystems and human health, causing air, soil, groundwater, and surface water pollution due to high concentrations of contaminants and methane production (Gautam et al. 2024) and will not aid in achieving the EU goals to reduce the landfill waste to 10% (EU 2018). The alternative approach is the integration of screening waste in the anaerobic digestion process together with other types of WWTP waste to ensure the energy recovery. However, despite the expected benefits of high organic matter content, energy production from screenings through anaerobic digestion faces hurdles caused by the presence of textiles and plastics (Cadavid-Rodriguez and Horan 2012) and results in low methane production yield (Boni et al. 2022; De la Torre-Bayo et al. 2022).

Nevertheless, the composition of screenings can still be regarded for bioenergy production. The main component of this type of waste is sanitary textiles, which can constitute more than 50% of the total volume and exhibit low coefficients of variation (De la Torre-Bayo et al. 2022). Knowing that this fraction is mainly composed of wipes and other hygiene products and considering the presence of paper and cardboard (11.8% and 10.52% of the volume), and vegetables (5.5% of the volume), the screenings can be characterized as a waste type with a high content of natural and regenerated cellulose (Ballesteros et al. 2022), thus making them a potential candidate for reuse through fermentable sugar production and biofuel generation.

Among the microorganisms, wood-decaying fungi are particularly renowned for their ability to degrade lignocellulosic biomass efficiently and thereby possess the potential for biological degradation of cellulose-rich waste. These fungi are capable of secreting a wide array of hydrolytic enzymes, such as cellulases and hemicellulases, and oxidative enzymes, including laccases, lignin peroxidases, and manganese peroxidases, necessary for breaking down lignin and ensuring complete lignocellulose degradation (Andlar et al. 2018). Fungi can thus serve as an alternative to physical and chemical treatment. Physical methods, like milling or steam explosion, enhance the accessibility of substrates for degradation but are energy-intensive. Chemical methods improve lignin breakdown but require constant chemical supply and tend to generate inhibitory compounds (Chatterjee and Mohan 2021). Biological pretreatments, particularly fungal-based methods, stand out as an eco-friendly and selective approach, requiring lower energy input and producing fewer harmful byproducts. This enhances both the environmental sustainability and economic feasibility of this technology. Moreover, the remarkable enzymatic diversity of fungi enables them to not only degrade lignin but also break down a wide range of xenobiotic compounds, including polycyclic aromatic hydrocarbons (PAHs) and chlorinated phenols (Dinakarkumar et al. 2024). Therefore, fungi not only play a crucial role in carbon cycling and organic matter decomposition across various ecosystems (Folman et al. 2008) but also can contribute to the degradation of environmental pollutants and lignocellulose-containing waste biomass. Fungal treatment could enable the valorization of WWTP-derived waste, preventing its disposal in landfills. This in turn would directly contribute to the reduction of methane emissions that primarily arise from the anaerobic decomposition of organic material (Karakurt et al. 2011), lower the risk of chemical leaching into the soil and water, and thus prevent environmental contamination.

Previously, the use of commercial cellulase and hemicellulose cocktails was reported for the enzymatic hydrolysis of cellulosic rejections recovered from a wastewater treatment plant in Barcelona (Ballesteros et al. 2022). The study reported the production of 29 g glucose and xylose per 100 g of the cellulosic rejections, which can subsequently be transformed into biofuels or bioproducts. However, the need for specific and expensive lignocellulose-degrading enzymes for efficient waste treatment could potentially limit the cost-effectiveness and use of this technology in the industrial scale.

Addressing the growing need for effective waste management and resource recovery, this study investigates the biodegradation potential and enzymatic behavior of white rot fungi, such as *Irpex lacteus*, *Bjerkandera adusta*, *Pleurotus dryinus*, and *Trametes versicolor*, in the biological treatment of WWTP-produced screenings, due to their ability to efficiently produce cellulolytic and ligninolytic enzymes (Civzele et al. 2023). This study proposes a novel approach for the sustainable treatment of cellulose containing-WWTP waste with limited reliance on expensive chemicals. In addition, the enzymatic behavior of selected fungi was also evaluated on lignocellulosic substrate to exclude the effect of potential pollutants found in the wastewater waste biomass. Moreover, additional research was conducted using lignin media to assess the fungal ability to produce oxidative or ligninolytic enzymes, which are applicable not only for lignin degradation but also for breaking down organic compounds and pollutants (Shanmugapriya et al. 2019) present in screenings. As a result, this study provides an analysis of the fungal enzymatic strategies and adaptability across different substrates, with findings that are applicable to the degradation of screening waste as well as various cellulose- and lignocellulose-containing biomass.

## Materials and methods

### Microorganisms

The following white rot fungi - *Irpex lacteus* DSM 9595, *Pleurotus dryinus* (Pers.) P. Kumm, *Bjerkandera adusta* DSM 23,426, and *Trametes versicolor* DSM 6401 were used in the study. The cultures were maintained on Potato Dextrose Agar (PDA) (Oxoid Ltd., Basingstoke, Hants, UK) medium at 2–8 °C.

### Substrates

Wastewater screening waste was collected after wastewater mechanical treatment in the biological wastewater treatment plant “Daugavgrīva” (Riga, Latvia, population equivalent of 700 000). The screenings used in this study were characterized by 19.78 ± 1.70% carbohydrate content.

The hay biomass (dry weight: 92.8 ± 1.3%; ash 6.03%) was collected from semi-natural grasslands in Latvia in 2022. The chemical composition of the hay biomass can be characterized by previous estimations of 22–26% cellulose, 14–25% hemicellulose, and 1–13% lignin (French 2019).

### Fungal cultivation conditions

Fungal cultures were cultivated in 250 mL Erlenmeyer flasks containing 150 mL of liquid medium consisting of 0.8 g KH_2_PO_4_, 0.4 g K_2_HPO_4_, 0.5 g MgSO_4_·7H_2_O, 2 g NH_4_NO_3_, 2 g yeast extract, and 10 g glucose per L, with the pH adjusted to 5.3–5.5. Cultures were incubated in an orbital shaker (New Brunswick™ Innova^®^ 43, Eppendorf Austria GmbH, Wien, Austria) at 150 rpm and 30 °C for 96 h until fungal pellets were formed.

### Experimental setup

Firstly, four types of liquid media were prepared, each with a base composition of 0.8 g KH_2_PO_4_, 0.4 g K_2_HPO_4_, 0.5 g MgSO_4_·7H_2_O, 2 g NH_4_NO_3_, and 2 g yeast extract per liter. The base media were further modified by adding 10 g of glucose per liter, or with 3% w/v lignin (Sigma-Aldrich, Darmstadt, Germany), hay as a lignocellulose representative, or wastewater screenings. All prepared media were autoclaved at 121 °C for 15 min to ensure sterility.

To initiate the fungal-assisted degradation experiments, freshly cultivated fungal cultures were washed using deionized water and inoculated in each type of media at a fungal biomass-to-substrate dry weight (DW) ratio of 1:20 to ensure a consistent amount of the inoculum across strains with differing growth characteristics. After inoculation, the cultures were incubated in an orbital shaker (New Brunswick™ Innova^®^ 43, Eppendorf Austria GmbH, Wien, Austria) at 30 °C and 150 rpm for 336 h. Flasks containing each medium type without the addition of fungi served as a negative control. Liquid media samples (1 mL) were collected daily. At the end of the cultivation experiments, the residual biomass was collected by filtration, washed with deionized water, and biomass samples were collected for analysis.

Each experiment was performed in three individual repeats. Standard deviations represent the data set from all repeats. Statistical significance of data was determined by unpaired t-tests with a significance threshold of *p* < 0.05. Unpaired t-tests were performed using GraphPad QuickCalcs (GraphPad Software, San Diego, CA, USA).

### Determination of total carbohydrates

The phenol-sulfuric acid method (DuBois et al. 1956) was used to determine the total carbohydrate concentration. For the analysis, 0.1 g of biomass samples (DW) were hydrolyzed by adding 3% sulfuric acid (H_2_SO_4_) and autoclaved at 121 °C for 20 min. The absorption of the resulting solution was measured using a UV-visible Spectrophotometer (GENESYS 150, Thermo Fisher Scientific Inc., Waltham, MA, USA) at a wavelength of 490 nm.

### Determination of reducing sugars

The concentration of reducing sugars was determined using the dinitrosalicylic acid (DNS) method (Ghose 1987). Within this study, the method was adjusted to microplate scale. In brief, 10 µL of the liquid sample was mixed with 10 µL of 0.05 M sodium citrate buffer and 60 µL of 3,5-dinitrosalicylic acid in the 96-well microplate. Distilled water was used as a control. All samples were heated at 100 °C for 5 min. Then, 220 µL of distilled water was added to each well, and the absorption of the solution was measured using the microplate reader (CLARIOstar plus, BMG Labtech) at 540 nm. To obtain absolute reducing sugar concentrations, the standard curve of known sugar concentration was constructed prior to reducing sugar analysis. The sugar concentration was defined as the mg of reducing sugars per g of dry biomass (mg/g).

### Fungal enzyme activity and total protein analysis

Ligninolytic enzyme activity was assessed in samples collected during the experiment. The samples were centrifuged at 8500 rcf for 5 min, and the obtained supernatant was then used in the assessment. The enzyme Units per mL (U/mL) of the sample were then converted to Units per mg of protein (U/mg).

#### Determination of total proteins

For protein quantification, the Bradford protein assay was used (Bradford 1976). 5 µL of liquid sample with the protein concentration in the range from 0.1 to 1.4 mg/mL was added to 96-well plate. In each well, 245 µL of Bradford reagent (Sigma, USA) was added, the plate was mixed at 300 rpm for 30 s and incubated for 5 min in the microplate reader (The CLARIOstar^®^ Plus, BMG Labtech, Germany). The absorption of the wells was measured at an absorption wavelength of 595 nm. 2 mg/mL protein standard (Sigma, USA) was used as a standard.

#### Cellulase assay

The fungal cellulolytic enzyme activity was analyzed by determining the *endo*-1,4-β-Glucanase (*endo*-cellulase) activity according to the colorimetric assay described in the Mangan et al. (2016) study. Following the assay protocol, the cellulase activity was determined by incubating the supernatant of the testing sample with a substrate solution containing β-glucosidase at 40 °C for 10 min. After the addition of the stopping reagent, 2% w/v Tris solution (pH 9), the absorbance of the resulting mixture was determined using a microplate reader (The CLARIOstar^®^ Plus, BMG Labtech, Germany) at 400 nm against the time zero reading for the respective substrate. One enzyme unit was defined as the amount of enzyme that released 1 µM of 4-nitrophenol from CellG5 per minute, units expressed as Units/mL.

#### Laccase assay

The fungal laccase activity was determined by the ABTS oxidation method (Wolfenden and Willson 1982). Oxidation of ABTS was measured by the determination of the increase in A_436_ (ε_436_ = 36000 M^− 1^·cm^− 1^). The reaction mixture contained 10 mM ABTS, 0.1 M sodium acetate buffer (pH 4.5), and the supernatant of the collected sample. Absorbance was measured using a microplate reader (The CLARIOstar^®^ Plus, BMG Labtech, Germany) at 436 nm against the distilled water as a blank. One enzyme unit was defined as the amount of enzyme that oxidized 1 µM of substrate per minute, units expressed as Units/mL.

#### Lignin peroxidase (LiP) assay

Lignin peroxidase (LiP) activity was measured according to the protocol described by Arora and Gill (2001). The analyzed mixture contained 120 µL sodium tartrate buffer (125 mM, pH 3.0), 60 µL Azure B (0.16 mM), 60 µL enzyme sample solution, 60 µL H_2_O_2_ (2 mM). The reaction started with the addition of H_2_O_2_ and was monitored using a microplate reader (The CLARIOstar^®^ Plus, BMG Labtech, Germany) at the absorption wavelength of 651 nm. One unit of enzyme activity is equivalent to an absorbance decrease of 0.1 units min^− 1^ ml^− 1^.

#### Manganese peroxidase (MnP) assay

Manganese peroxidase (MnP) activity was determined according to Arora et al. (2002). Within this study, the method was adjusted for microplate scale. The reaction mixture contained the collected sample solution, 50 mM sodium succinate buffer (pH 4.5), 50 mM sodium lactate (pH 5), 0.1 mM MnSO_4_, 0.1 mM phenol red, and 100 µM H_2_O_2_. The reaction was initiated by addition H_2_O_2_ and conducted at 30℃ (ThermoMixer^®^ C, Eppendorf^®^, Germany). After 10 min, 100 µL of reaction mixture was taken, and 4 µl of 5 N NaOH was added to it. Absorbance was measured using a microplate reader (The CLARIOstar^®^ Plus, BMG Labtech, Germany) at 610 nm. One unit of enzyme activity is equivalent to an absorbance increase of 0.1 units min^− 1^ ml^− 1^.

## Results

### Fungal enzymatic activity in optimal conditions

Glucose as a carbon source was used to determine the enzymatic activity profile of the selected white rot fungi under optimal cultivating conditions with readily available sugars and without lignocellulosic biomass. In the absence of more complex carbon compounds, using only easily available sugars, rapid and high-yield fungal biomass production was achieved. At the early stage of the experiment, glucose consumption and relatively low enzyme activity were observed. After 72 h, glucose concentration decreased by more than 40% in all samples with fungi. In samples supplied with *T. versicolor*, the concentration decreased by 80%. This corresponded to an active growth of fungal biomass with a total increase of 3.27 times from the initial dry weight of the fungal inoculum within the total incubation time of 336 h.

After 240 h, when glucose was utilized, a subsequent increase in enzyme activity was observed (see Additional file 1, Fig.S1-S2). For *I. lacteus*, only a low concentration of MnP and LiP was observed within the first 72 h of cultivation. After 168 h of cultivation, when the reducing sugar concentration was below 100 mg/g or reached 10% of the initial concentration, an increase in LiP activity was observed, and a significant peak in LiP (19.14 ± 3.05 U/mg) emerged after 264 h. In contrast, MnP concentration exhibited no significant alterations during this period. Laccase and cellulase were not detected in any of the *I. lacteus* replicates when cultivated in glucose-containing media. *B. adusta* showed an increase in LiP activity in the first cultivation hours, followed by a decrease and stabilization at around 1 U/mg after 72 h. *B. adusta* exhibited an increase in laccase activity after 288 h, around 48 h after complete glucose uptake, reaching a peak at 336 h (258.62 ± 61.04 U/mg). No cellulase activity and no significant MnP activity were detected in any of the *B. adusta* replicates.

No cellulase activity was also detected in *P. dryinus* replicates, and peroxidase activity remained below 1 U/mg throughout the cultivation. In contrast, laccase activity remained around 100 U/mg or less throughout the whole cultivation period. At the same time, after 24 and 144 h of incubation, significant peaks of laccase production were detected for *T. versicolor* − 225.93 ± 121.42 and 236.18 ± 122.27 U/mg, respectively. Additionally, after 144 h, MnP activity of 14.74 ± 11.17 U/mg was observed. The rise of enzyme activity after 144 h corresponds to complete glucose uptake by fungi; however, LiP and cellulase activity were not detected in any of the *T. versicolor* replicates.

The absence of enzymatic activity in white rot fungi in the presence of available sugars corresponds to a phenomenon known as carbon catabolite repression (CCR) (Adnan et al. 2017; de Assis et al. 2021), a regulatory mechanism that prioritizes glucose utilization while repressing the expression of genes involved in the degradation of alternative carbon sources. In ascomycetes, CCR is primarily mediated by the transcriptional repressor CRE1/*CreA*, which is triggered by monomeric sugars (Hu et al. 2021; Rytioja et al. 2014). A similar mechanism exists in basidiomycetes, where *CreA* homologs have been identified (Rytioja et al. 2014). In *T. versicolor*, *CreA*-like binding sites have been observed in the cellobiose dehydrogenase gene, indicating involvement in CCR (Stapleton and Dobson 2003). In *P. ostreatus*, overexpression of CRE1 reduced cellulolytic activity, while its knockout led to enhanced secretion of cellulolytic and other carbohydrate-active enzymes (Yoav et al. 2018). Following complete glucose uptake, fungi shift from active fungal biomass growth and primary metabolism to the initiation of the secondary metabolism stage (Avalos and Limón 2021), promoting the secretion of lignocellulolytic enzymes.

### Fungal activity in lignocellulose-containing media

**Fig. 1:**
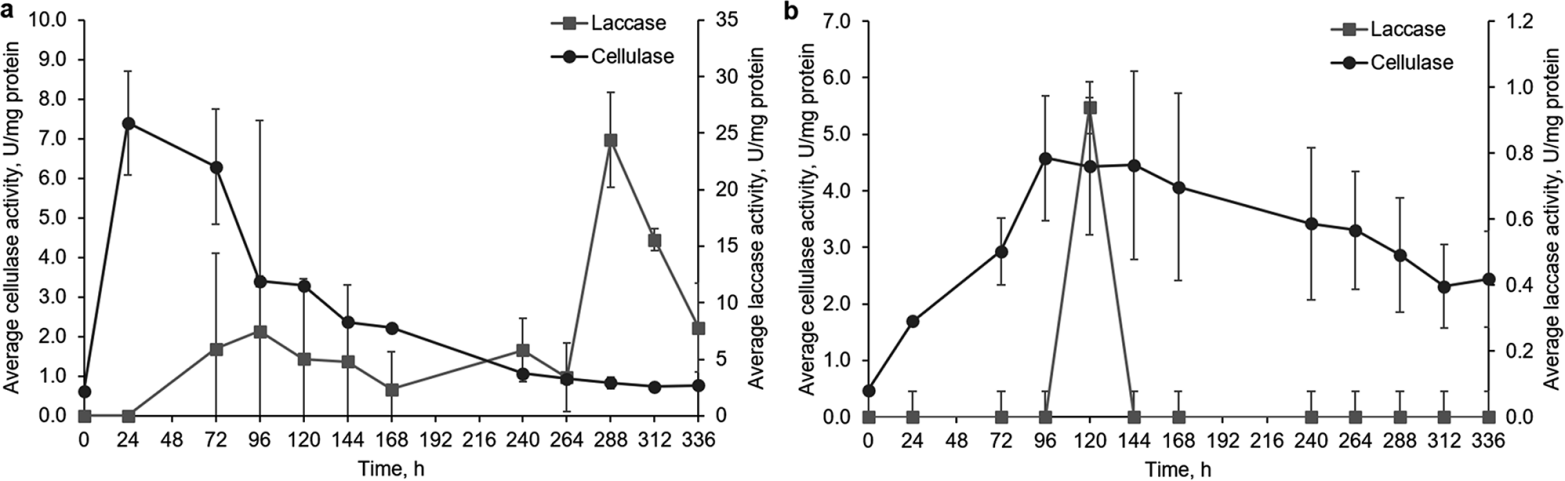
The activity of cellulase and laccase in (**a**) *I. lacteus* and (**b**) *B. adusta* cultures cultivated in the hay biomass – containing media. Error bars represent the standard deviation of the three replicates

The cultivation in media containing hay biomass without glucose led to elevated expression of cellulolytic and ligninolytic enzymes in all studied white rot fungi. *I. lacteus* exhibited a peak in cellulase production, reaching a maximum activity of 7.40 ± 1.30 U/mg after 24 h of incubation, and laccase activity peaked at 288 h (24.40 ± 4.21 U/mg) (Fig. 1). The observed enzyme kinetics align with the characteristics of lignocellulosic biomass and pretreatment methods. At the beginning of the incubation period, simple sugars are readily available in the medium due to thermal pretreatment, providing an immediate energy source for fungal biomass growth. Additionally, mechanical pretreatment (milling) reduces particle size, improving accessibility to cellulose, which facilitates early cellulase secretion. As incubation progresses and the readily available sugars are consumed, fungi shift their metabolic strategy toward more complex compound degradation. This transition leads to a gradual increase in ligninolytic enzyme production, with peak laccase activity observed around 96 h, coinciding with the consumption of simpler carbon sources.

*B. adusta* showed an increase in cellulase activity, with a peak activity at 96 h and a steady decrease afterwards. The observed increase in enzymatic activity during the last 24 h of cultivation suggests that *B. adusta* holds potential for continued waste degradation beyond the 336-hour incubation period. After 120 h, a peak laccase activity of 0.938 ± 0.26 U/mg (*p <* 0.05) was observed. However, *I. lacteus* peak laccase activity is considered significantly higher than the peak activity of *B. adusta.* Additionally, in both *I. lacteus* and *B. adusta* replicates, no significant LiP and MnP activity was detected.

**Fig. 2:**
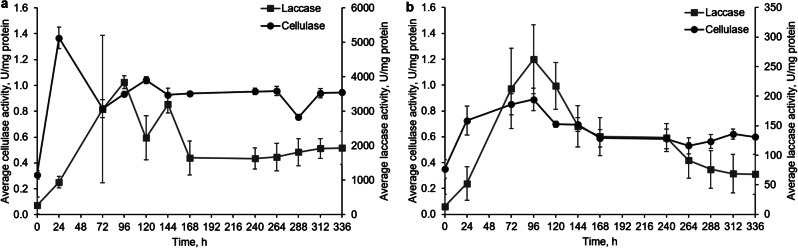
The activity of cellulase and laccase in (**a**) *P. dryinus* and (**b**) *T. versicolor* cultures cultivated in the hay biomass – containing media. Error bars represent the standard deviation of the three replicates

In contrast, cellulase activity in both *P. dryinus* and *T. versicolor* was significantly lower than in *I. lacteus* and *B. adusta* (Fig. 2). *P. dryinus* showed a cellulase peak of only 1.37 ± 0.08 U/mg at 24 h (*p <* 0.05), followed by a decrease and stabilization at 1.0 U/mg for the remaining incubation period. Cellulase activity of *T. versicolor* increased from 0.35 ± 0.07 to 0.89 ± 0.09 U/mg in the first 96 h of incubation with a significant peak at 96 h (*p* < 0.05) and subsequently declined. Even though the peak values are significantly lower than in *I. lacteus* and *B. adusta*, each peak is a significant increase for each fungus. The observed laccase activity in both *P. dryinus* and *T. versicolor* was up to 100 times higher than the laccase activity of *I. lacteus* and *B. adusta*. Two peaks of laccase production were detected in *P. dryinus* at 96 and 144 h (3845.15 ± 190.92 and 3202.52 ± 247.48 U/mg). In *T. versicolor*, laccase activity increased, reaching a highest activity of 262.43 ± 58.33 U/mg after 96 h of incubation.

When lignocellulose-containing media is used, enzymatic activity in all examined fungi begins by secretion of cellulolytic enzymes to degrade the available cellulose. Relatively low laccase activity combined with stable cellulase activity shows that *B. adusta* and *I. lacteus* produced predominantly cellulolytic enzymes, which were able to provide the necessary carbon with less significant expression of ligninolytic enzymes. At the same time, the secretion of the ligninolytic enzymes was observed in *P. dryinus* and *T. versicolor* that indicated the potential breakdown of lignin polymers and more comprehensive degradation of the complex lignocellulosic matrix. The activity of other analyzed ligninolytic enzymes (MnP and LiP) in *P. dryinus* remained under 1 U/mg during the incubation time. A significant increase in LiP and MnP activity was observed at 120 h and 144 h, respectively. For *T. versicolor* no significant change in the peroxidase activity was detected, and it remained in the range from 0.2 to 0.4 U/mg throughout the incubation period. These low concentrations suggest that the main ligninolytic enzyme in *T. versicolor* lignocellulosic biomass degradation was laccase.

**Fig. 3:**
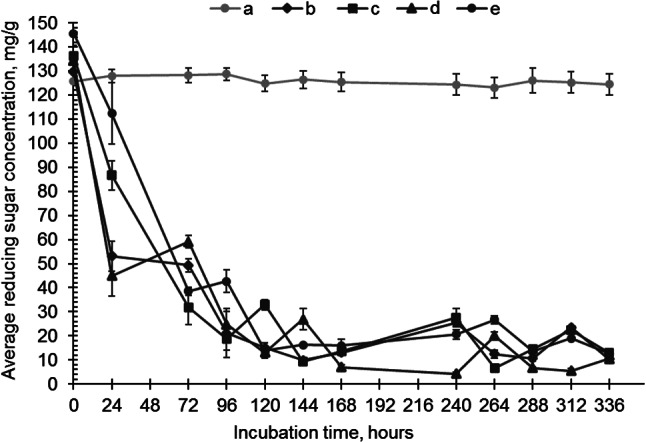
The concentration of reducing sugars in **a**) control, **b**) *I. lacteus*, **c**) *P. dryinus*, **d**) *B. adusta*, and **e**) *T. versicolor* culture cultivated in the hay biomass – containing media. Error bars represent the standard deviation of the three replicates

As a consequence of the observed lignocellulolytic enzyme activity, a decrease in carbohydrate content and reducing sugar production was observed in all samples with added fungi (Fig. 3). All studied fungi first utilized the readily available sugars, reducing the available sugar concentration by 19–32% within the first 24 h. Subsequently, in samples with *I. lacteus*, the reducing sugar concentration gradually increased, beginning with 144 h (9.83 ± 0.60 mg/g), peaking at 240 h (25.50 ± 1.14 mg/g), and later at 312 h (23.51 ± 0.58 mg/g). While utilizing the available carbon, *I. lacteus* simultaneously produced cellulolytic enzymes, and the observed rise in sugar corresponds with the increase in cellulase activity after 120 h, performing both cellulose degradation and sugar release. In samples with *B. adusta*, a cyclic pattern with an initial sugar concentration reduction was observed - the highest sugar yield at 72 h (59.09 ± 2.46 mg/g), a decline and subsequent increase at 144 (26.86 ± 4.42 mg/g) and 264 h (20.02 ± 1.70 mg/g). This can be explained by the active cellulolytic enzyme production observed throughout the study.

*P. dryinus* produced 32.88 ± 2.18 mg reducing sugars per g substrate after 120 h as a result of cellulose degradation ensured by previously secreted cellulases. Also, for *T. versicolor*, a similar reducing sugar production pattern was observed, as the sugar concentration gradually increased after 120 h (13.84 ± 1.47 mg/g) and reached the peak at 264 h (26.64 ± 1.74 mg/g) and 312 h (19.03 ± 0.50 mg/g). This gradual production corresponds with the observed peaks of cellulase and laccase activity secreted by *T. versicolor* after 96 h of incubation.

The initial carbohydrate content in hay was 48.71 ± 1.43% (equivalent to 487.06 ± 14.27 mg of carbohydrates per g of biomass). Treatment with fungi resulted in a reduction of the overall carbohydrate content ranging from 34.60 to 51.54%. The most substantial decrease in carbohydrate content occurred in samples with *P. dryinus*, where the resulting biomass contained only 23.6 ± 1.06% total carbohydrates. *I. lacteus* and *B. adusta* also significantly reduced the carbohydrate content in the treated lignocellulosic biomass, resulting in remaining carbohydrate levels of 25.05 ± 2.97% and 28.47 ± 1.52%, respectively. The lowest impact on the carbohydrate content was observed for *T. versicolor*, which nonetheless demonstrated the significant degradation capability, reducing the carbohydrates by nearly 35%. Moreover, all fungi demonstrated active biomass growth when cultivated on lignocellulosic biomass. Despite the more complex conditions compared to glucose-containing media, the fungal mass increase in samples with hay was only 8% less than under optimal conditions. This suggests that the fungi exhibit significant adaptability, allowing them to grow nearly as well on lignocellulosic substrates, which closely resemble their natural environment, as they do under optimal conditions.

### Fungal activity in WWTP-derived screenings

**Fig. 4:**
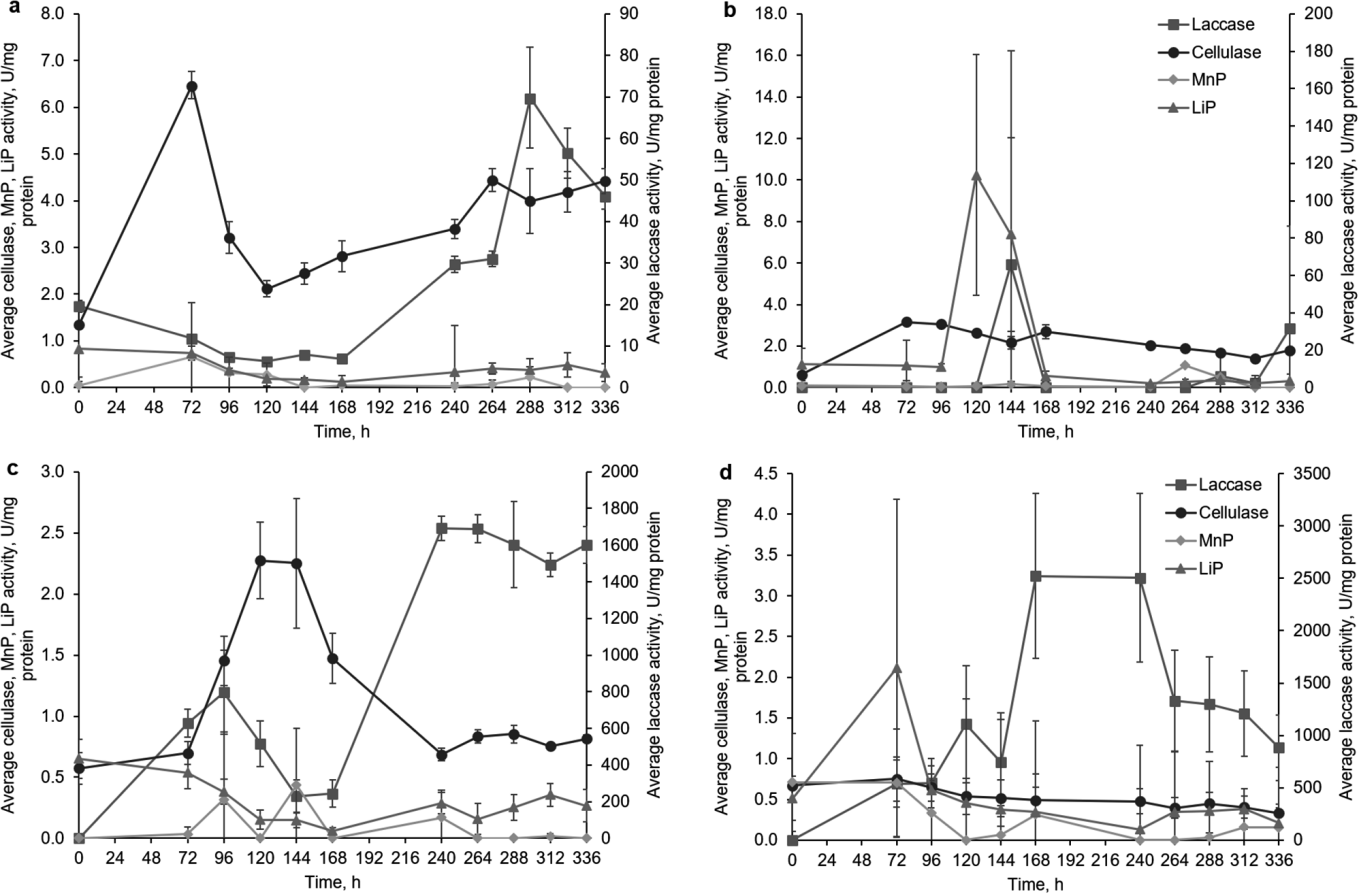
The activity of cellulase, manganese peroxidase (MnP), lignin peroxidase (LiP) and laccase in (**a**) *I. lacteus*, (**b**) *B. adusta*, (**c**) *P. dryinus* and (**d**) *T. versicolor* cultures cultivated in the WWTP- derived screenings – containing media. Error bars represent the standard deviation of the three replicates

The presence of screenings as a cellulose-containing feedstock and the sole carbon source during cultivation resulted in the secretion of cellulase in all tested white rot fungi (Fig. 4). Moreover, *I. lacteus* exhibited laccase activity, which was two times higher than observed during cultivation with hay biomass. Higher laccase activity was also detected in *B. adusta*, *P. dryinus*, and *T. versicolor* samples. All studied white rot fungi also displayed instability in enzyme production with rapid enzyme activity fluctuations throughout the cultivation period.

*I. lacteus* exhibited a cellulase activity peak within the first 72 h of cultivation, reaching a maximum value of 6.48 ± 0.30 U/mg. Concurrently, ligninolytic enzyme activity declined during this period. After 120 h, cellulase activity reached its lowest value, followed by a steady increase in the laccase activity. Another notable increase occurred after 264 h of cultivation (4.44 ± 0.25 U/mg) with a subsequent increase in laccase activity (69.84 ± 12.22 U/mg) at 288 h. Following this peak in laccase activity, cellulase activity exhibited a continuous increase.

Similarly to the enzymatic activity of *I. lacteus* observed in hay-containing media, the cellulases acted first by degrading the available cellulose in the screenings and were followed by an increase in the laccase activity. Cellulase activity in both screenings and hay biomass samples was similar; however, laccase activity was significantly higher when *I. lacteus* was cultivated in samples with screenings, potentially responding to the presence of organic pollutants or other complex compounds. Both peroxidases displayed no significant activity, with the values remaining under 1 U/mg and with no rapid fluctuations, suggesting that the main enzymes involved in the waste treatment with *I. lacteus* are cellulases and laccase.

When subjected to screenings, *B. adusta* demonstrated a consistent increase in cellulase activity within the initial 72 h of cultivation, followed by a subsequent decrease, as also seen in *I. lacteus* cultures. Only minor deviations from 2 to 2.5 U/mg were observed during this decrease. At the same time in the time period from 72 to 144 h, peaks in LiP and laccase were identified (10.23 ± 5.80 U/mg at 120 h and 65.82 ± 114.33 U/mg at 144 h, respectively). Following these ligninolytic enzyme peaks, an increase in cellulase activity was registered. After 336 h of cultivation, another laccase peak of 31.67 ± 54.90 U/mg was detected, accompanied by a simultaneous rise in cellulase activity.

*P. dryinus* displayed a different enzymatic mechanism for the degradation of screenings than *I. lacteus* and *B. adusta*. First, a steady rise in laccase activity to a maximum of 796.84 ± 228.90 U/mg after 96 h was observed. Then an increase in cellulase activity began, suggesting the availability of additional cellulose material subsequent to ligninolytic enzyme secretion and potential lignin degradation. A rapid increase in cellulase activity within 72 to 120 h was registered, with a peak value of 2.27 ± 0.31 U/mg. Then the cellulase activity decreased, and at 240 h a second peak in laccase activity was detected (1691.75 ± 12.22 U/mg) with a subsequent increase in cellulase activity. The enzyme dynamics during 168 to 240 h show the waste degradation patterns in *P. dryinus* with cellulase and laccase degrading the waste biomass subsequently. Additionally, a simultaneous increase in LiP activity was detected from 264 to 312 h of cultivation. However, it is noteworthy that peroxidase activity remained consistently under 1 U/mg throughout the entire cultivation period.

*T. versicolor* also displayed a high laccase activity with several recorded peak values, similarly to *P. dryinus*. After 120 h of incubation, a peak value of 1108.56 ± 558.52 U/mg was recorded (*p* > 0.05), then followed by a peak of 2521.15 ± 787.72 U/mg (*p* < 0.05). Unlike other studied white rot fungi, the increase in laccase secretion in *T. versicolor* was not followed by an increase in cellulase activity. The results suggest that in the treatment process of screenings mediated by *T. versicolor*, the dominant enzyme was laccase, mirroring the outcomes obtained in lignocellulose biomass degradation.

**Fig. 5:**
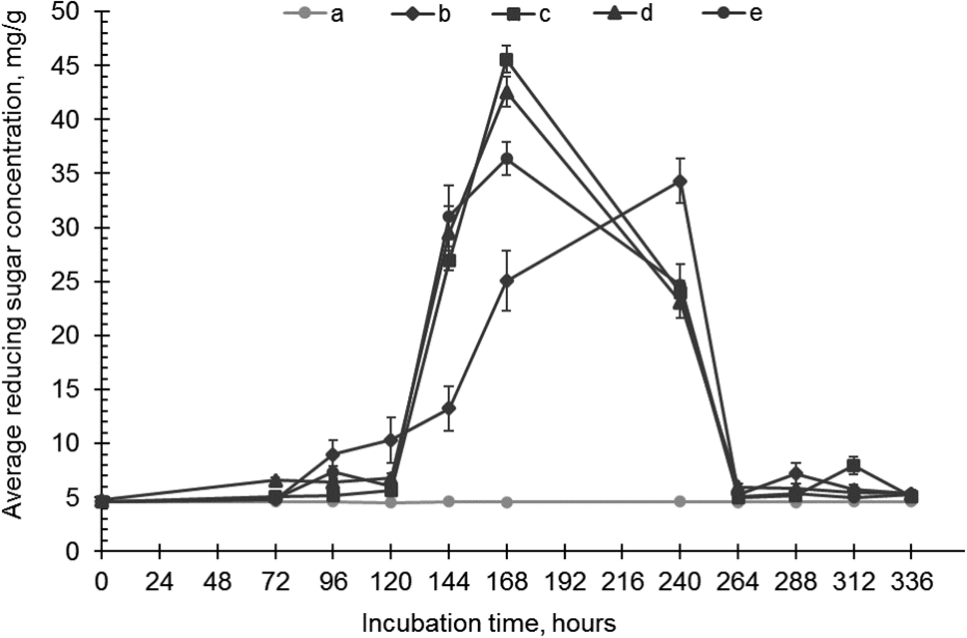
The concentration of reducing sugars in (**a**) control, (**b**) *I. lacteus*, (**c**) *P. dryinus*, (**d**) *B. adusta*, and (**e**) *T. versicolor* culture cultivated in the WWTP- derived screenings – containing media. Error bars represent the standard deviation of the three replicates

When cultivated in WWTP-derived screenings, no significant changes in sugar concentration were detected during the initial 48–72 h of incubation (Fig. 5). However, after 96 h, the first increase in reducing sugar concentration was observed in samples with *I. lacteus* (8.98 ± 1.33 mg/g) and *T. versicolor* (7.33 ± 0.58 mg/g). *I. lacteus*, which had a shorter adaptation time, facilitated an increase in reducing sugar concentration for 96 h, reaching a maximum after 240 h (34.32 ± 2.03 mg/g), then rapidly declined. On the other hand, *P. dryinus*, *B. adusta*, and *T. versicolor* exhibited a rapid increase in sugar concentration after 120 h, and the peak of sugar production by these fungi was observed at 168 h – 45.57 ± 1.25 mg/g, 42.55 ± 1.40 mg/g, and 36.36 ± 1.52 mg/g, respectively.

During the first 120 h, when the initial sugar concentration was relatively low to provide the necessary feedstock, all fungi exhibited elevated enzymatic activity, indicating on an adaptation period to the substrate. This phase led to the observed production of reducing sugars, essential for the metabolic needs of the studied fungi, which were subsequently utilized. Also, despite the limited carbon source and the potential inhibitory effects of the pollutants, fungal biomass growth in screenings reached 52% of the biomass growth efficiency observed in glucose media.

Moreover, after 336 h of incubation, all fungi had a significant impact on the total carbohydrate content in the screenings. Similar to the results observed for lignocellulosic biomass degradation, *P. dryinus* and *B. adusta* showed the most substantial reductions in carbohydrate content, decreasing it from 19.78 ± 1.70% to 10.99 ± 1.08% and 11.18 ± 1.06%, respectively. In samples with I. *lacteus* and *T. versicolor*, the decrease in carbohydrates also occurred; these reached 12.11 ± 1.44% and 12.61 ± 2.08%, respectively.

### Fungal enzymatic activity in lignin-containing media

To investigate the fungal adaptability and enzyme strategies for breaking down and utilizing lignin, as well as potential organic compounds often found in the WWTP-derived waste, the enzymatic behavior of the fungi during the lignin biomass treatment was also studied. The fungal cultivation in lignin-containing media demonstrated significantly lower enzymatic activity than in other substrates (see Additional file 1, Fig.S3). No significant increase in MnP or LiP activity was detected in any of the replicates for all white rot fungi, the activity remained under 2 U/mg. *I. lacteus* showed no increase in laccase activity, which remained at the baseline under 3 U/mg. *P. dryinus* peaked in laccase activity, reaching the highest value after 120 h of incubation. However, the peak is not considered significant (*p* > 0.05) compared to the enzyme activity at 96 and 144 h of incubation. No cellulase activity was detected in any of the replicates for all white rot fungi due to the absence of cellulose in the culture media. Additionally, in lignin media, fungal biomass increased only by 18 to 34% after 336 h of incubation. This suggests that lignin degradation products were not suitable for enhanced fungal biomass growth.

## Discussion

Both lignocellulose and screenings from primary wastewater treatment contain significant amounts of lignocellulosic material that can be degraded by white rot fungi and converted into fermentable sugars. Given the established role of white rot fungi in lignocellulose biomass degradation, these microorganisms exhibit a significant potential for waste treatment (Bogale 2020; Atiwesh et al. 2022). However, the primary difference lies in the complexity and variability of the screenings, which often include plastics, fine particles, and potential contaminants (De la Torre-Bayo et al. 2022) not found in hay or the other lignocellulosic biomass. While hay represents a controlled and well-defined lignocellulose source, screenings, derived from WWTPs, introduce real-world complexity with potential contaminants. These impurities in screenings could pose additional challenges for fungal activity and enzymatic degradation. Screenings typically consist of a heterogeneous mix of organic and inorganic materials, including plant debris, synthetic fibers, plastics, fats, and various contaminants. On one hand, the presence of recalcitrant or toxic compounds can inhibit fungal growth or enzyme expression. On the other hand, the diverse organic components may serve as a source of carbon, potentially stimulating the enzymatic production. Therefore, the selection of robust fungal strains capable of adapting to such complex substrates is critical.

Among the studied white rot fungi, variable responses to the substrates and different enzyme production strategies were observed during the cultivation in both lignocellulose and WWTP-derived waste biomass. Nevertheless, a significant fungal biomass increase across all types of media indicates the high adaptability of the selected strains to complex substrates and challenging conditions. Depending on the structure and composition of the waste biomass, a cellulolytic or ligninolytic fungal strain could be more favorable. All fungi demonstrated active production of both hydrolytic and oxidative enzymes while growing in screened waste-containing media and were capable of converting carbohydrates present in waste into fermentable sugars.

The degradation of lignocellulosic substrate that is a natural environment for white rot fungi began within the first 72 h. Whereas sugar production in screenings appeared after 96 h for *I. lacteus* and after 120 h for *P. dryinus*, *B. adusta*, and *T. versicolor.* The longer adaptation period highlights the potential effects of the lower concentration of available carbohydrates and the potential presence of inhibitory substances that may have delayed the fungal metabolism and the breakdown of lignocellulosic compounds.

In both lignocellulose substrate and screenings, all fungi exhibited the degradation of at least 34% of total carbohydrates, while the highest biomass conversion efficiency was demonstrated by *P. dryinus*. This fungus was able to degrade over 51% of carbohydrates detected in the hay biomass and nearly 45% of carbohydrates in screenings. Moreover, *P. dryinus* showed the highest reducing sugar concentration (45.57 ± 1.25 mg/g biomass) during the incubation in WWTP-derived waste, representing nearly 52% of degraded carbohydrate volume. The remaining carbohydrates may have been utilized by the fungus to support its metabolism and enzymatic activity.

A significant screening degradation efficiency was also detected in *B. adusta*. While reducing the carbohydrate levels in lignocellulosic biomass by 41.50%, this fungus achieved up to a 43.50% reduction in total carbohydrates in screenings. Furthermore, 49.47% of degraded carbohydrate volume was comprised of reducing sugars. Nearly equivalent efficiency in the carbohydrate degradation in both screenings and lignocellulosic biomass was also demonstrated by *T. versicolor*, where over 36% and 34.50% of carbohydrates were degraded, respectively. Moreover, after 120 h of incubation in WWTP-derived waste, *T. versicolor* also produced fermentable sugars, which accounted for more than 50% of the converted carbohydrates. At the same time, among the studied fungi, *I. lacteus* exhibited the highest difference in degradation efficiency between the waste and lignocellulose substrates. While degrading more than 48% of carbohydrates in hay, degradation of nearly 39% of carbohydrates was observed in screenings. However, 44.77% of these carbohydrates were determined as reducing sugars, still showing a potential for the WWTP-generated waste biomass bioconversion for biofuel production. Based on the total carbohydrate content in the screenings and the observed saccharification rates, it is possible to estimate the ethanol yield by considering the conversion of reducing sugars to ethanol. Specifically, 1 kg of screenings would result in 27.80 mL of ethanol when treated with *P. dryinus* and 29.70 mL of ethanol when treated with *B. adusta*.

While exhibiting similar efficiency in lignocellulose conversion, white rot fungi exhibited completely different enzymatic behavior if screenings were used as the feedstock. The metabolic strategy of *P. dryinus* was based on the high initial activity of laccases, peaking at 96 h with a notable value of 796.84 ± 228.90 U/mg. After this peak, cellulase activity began to increase significantly, indicating that once the more complex, lignin-like structures had been partially degraded, *P. dryinus* shifted its focus towards cellulose breakdown and sugar production. An increase in LiP activity observed from 264 to 312 h, while peroxidase activity remained consistently low throughout the cultivation period, suggests that *P. dryinus* primarily uses laccase for the oxidative processes and may utilize LiP for further degradation processes in more extended cultivation periods.

In contrast, *B. adusta* exhibited a different enzymatic approach, demonstrating the steady rise in cellulase activity during the initial 72 h, followed by a gradual decline with fluctuations around 2 to 2.50 U/mg. This consistent cellulase activity suggests a strong initial focus on cellulose degradation, which is essential for breaking down the primary polysaccharides in lignocellulosic biomass. During the decline in cellulase activity, peaks in lignin peroxidase (LiP) and laccase activities were observed at 120 and 144 h. These peaks suggest that *B. adusta* shifts its enzymatic focus from cellulose to oxidative processes demanding ligninolytic enzyme production after an initial period of cellulase activity.

Despite the lower reducing sugar production, *T. versicolor* showed a predominant focus on laccase production, with significant peaks in enzyme activity observed throughout the cultivation period, similarly to *P. dryinus*. However, *T. versicolor* did not exhibit a corresponding increase in cellulase activity following laccase peaks. This suggests that *T. versicolor* prioritized oxidative enzyme production and complex compound degradation over cellulose conversion, possibly due to the complex nature of the screenings and its adaptability to these conditions.

*I. lacteus* had the shortest adaptation time to initiate carbohydrate degradation, however, had the latest peak of sugar production yield. This fungus employed cellulase production during the initial 72 h to degrade cellulose, followed by a notable increase in laccase activity as cellulose becomes more available. This transition suggests that *I. lacteus* similarly to *B. adusta* first targets the accessible cellulose for conversion, then shifts to the ligninolytic enzyme secretion in response to the presence of organic pollutants.

Despite the compositional differences between lignocellulose and WWTP screenings and the variable adaptation time needed, after 336 h, the carbohydrate degradation efficiency in both substrates was comparable. Notably, fungi like *T. versicolor* and *B. adusta* exhibited even higher efficiency in the environment with complex material, such as screenings. Moreover, cultivation in screenings boosted the enzymatic activity in all studied fungi. The response to the complex substrate and the potential presence of organic compounds led to substantial ligninolytic enzyme activity in *P. dryinus* and *T. versicolor*, which exhibited almost 1700 U/mg and more than 2500 U/mg laccase activity, respectively, during the WWTP waste treatment. This exceptional capability to secrete ligninolytic enzymes makes *P. dryinus* and *T. versicolor* strong candidates not only for lignin-rich biomass degradation but also for the enzymatic oxidation of organic compounds in wastewater and waste treatment. The study also demonstrates that fungal-assisted degradation of WWTP-derived waste can result not only in fermentable sugar production, promoting sustainable waste disposal and bioenergy production, but also in the production of hydrolytic and oxidative enzymes with remarkable activity. After degrading screened waste and producing sugars for biofuel, these enzymes can potentially be extracted and used repeatedly or for other biotechnological applications.

Therefore, in future studies, the potential of the described fungal-based waste treatment approach, as well as the mechanisms behind the enzymatic responses to potential inhibitory factors, should be further investigated. The development of this waste treatment method, with the integration of white rot fungi, requires optimizing fungal cultivation conditions and potential pretreatment methods to enhance fermentable sugar yields. In case of more complex waste or biomass treatment, the application of several fungal strains in co-cultivation conditions or the production of fungal enzymes and the use of enzyme cocktails should be investigated. Cellulolytic fungi, with high cellulase activity, are beneficial for bioethanol production and composting, where efficient cellulose breakdown is essential. In contrast, the integration of ligninolytic fungi is valuable in bioremediation, wastewater treatment, and biomass pretreatment for biofuels. Even in the absence of cellulase activity, fungal biodelignification can enhance biomass digestibility for downstream processing, making strain selection crucial for specific applications. Additionally, evaluating the economic feasibility and scalability of fungal-assisted treatment of screenings is necessary to advance this technology towards practical application.

The observed inhibition of fungal enzymatic activity in lignin-rich media shows the need for further research on the interactions between pure lignin and fungal enzymes. Interactions and effects of different biomass pretreatment and lignin removal technologies as well as the characteristics of lignin on the enzymatic activity have been described before, however, there is limited research on enzyme secretion in the presence of pure lignin. Moreover, lignin residues and soluble lignin-derived phenolic compounds can have an inhibitory effect and inactivate secreted enzymes (Pamidipati and Ahmed 2020). Although white rot fungi, particularly *P. dryinus* and *T. versicolor*, can produce highly active ligninolytic enzymes, their ability to degrade pure lignin under carbon-limited conditions showed to be limiting. Unlike their robust response to cellulose-rich substrates, the fungi showed significantly lower biomass growth and reduced enzymatic activity in the presence of lignin, with laccase activity decreased and manganese peroxidase (MnP) and lignin peroxidase (LiP) activity remaining below 2 U/mg. This suggests the necessity of mixed-substrate environments to effectively stimulate comprehensive lignocellulolytic enzyme production. Additionally, ligninolytic enzyme activity can be inhibited by pH, temperature, high carbon, or nitrogen content, as well as higher heavy metal or halide concentrations (Paterson et al. 2008). Another inhibiting factor for laccase is the competitive inhibition of laccase by lignin degradation intermediates – fungal-solubilized lignin and alkali-treated lignin (Pamidipati and Ahmed 2020). Reversing these negative effects could involve inducing enzyme expression with low concentrations of glucose or any of the known inducers, such as veratryl alcohol and Cu^2+^ (Vasconcelos et al. 2000; Levin et al. 2008), thereby promoting the transcription of laccase, LiP, and MnP (Krumova et al. 2018). Future studies could focus on these strategies or consider co-degradation approaches where lignin is treated alongside lignocellulosic biomass to enhance degradation efficiency. To treat pure lignin biomass independently, enzyme production by the fungi should precede the treatment, or pure lignin can be subjected to treatment using previously secreted and purified ligninolytic enzymes alone.

## Conclusions

The study shows the potential of white rot fungi, particularly *P. dryinus* and *B*. *adusta*, for the biological treatment of WWTP-derived screenings. These fungi demonstrated the effective degradation of complex substrates, reducing carbohydrate content by over 43.50% and producing more than 42 kg of fermentable sugars per tonne of screenings. All studied fungi were able to adapt and grow in WWTP waste media and were capable of producing highly active hydrolytic and oxidative enzymes. The most substantial enzymatic activity was observed in *P. dryinus* and *T. versicolor*, which secreted laccases with nearly 1700 U/mg and over 2500 U/mg enzymatic activity, respectively. As a result, the integration of white rot fungi presents a sustainable approach to reduce the impact of WWTP-generated waste and ensure an eco-friendly valorization route for waste screened during the preliminary wastewater treatment. Moreover, the biological degradation of screenings can result not only in sugar production and bioenergy generation but also in the production of high-value chemicals for other biotechnological applications. Therefore, further research is needed to optimize fungal cultivation, explore pretreatment methods, and address potential inhibitory factors for industrial scalability.

## Electronic supplementary material

Below is the link to the electronic supplementary material.

Supplementary Material 1

## Data Availability

No datasets were generated or analysed during the current study.
