# Supplementary material for: Biodegradation of screenings from sewage treatment by white rot fungi
Source: Fungal Biol Biotechnol. 2025 May 14;12:7. doi: 10.1186/s40694-025-00198-5 (PMC12080140; doi:10.1186/s40694-025-00198-5)
Supplement: Supplementary file 1 — Supplementary Material 1 [file 40694_2025_198_MOESM1_ESM.pdf]

Supplementary material

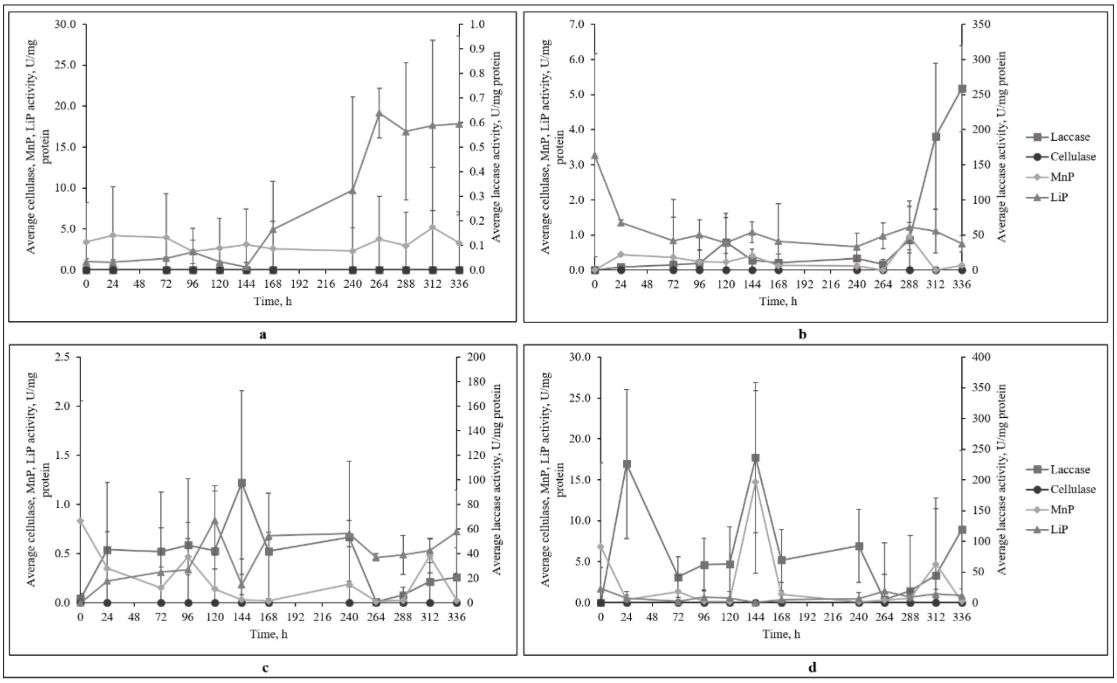

Figure S1. The activity of cellulase, manganese peroxidase (MnP), lignin peroxidase (LiP) and laccase in (a) *I. lacteus*, (b) *B. adusta*, (c) *P. dryinus* and (d) *T. versicolor* cultures cultivated in the glucose – containing media.

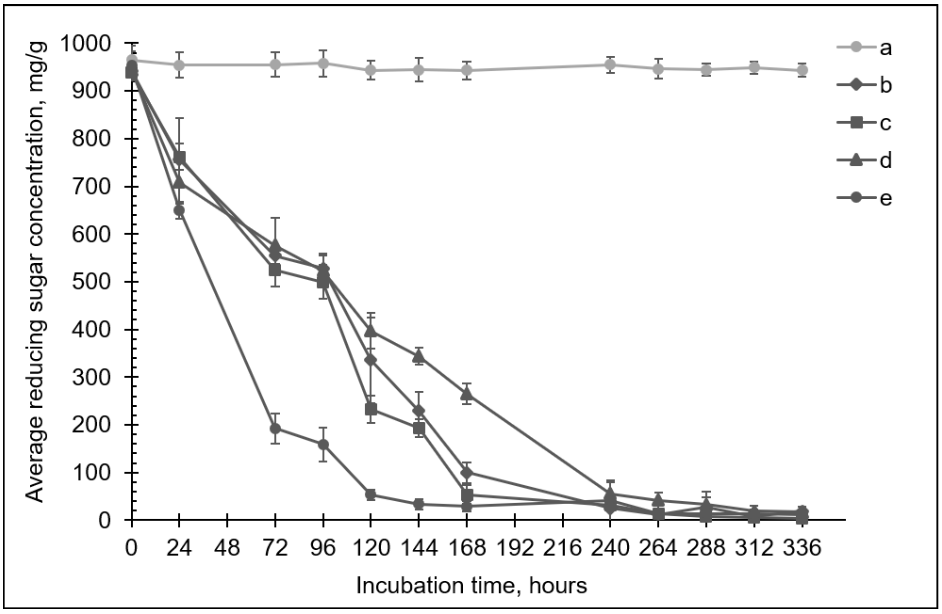

Figure S2. The concentration of reducing sugars in a) control, b) *I. lacteus*, c) *P. dryinus*, d) *B. adusta*, and e) *T. versicolor* culture cultivated in the glucose – containing media.

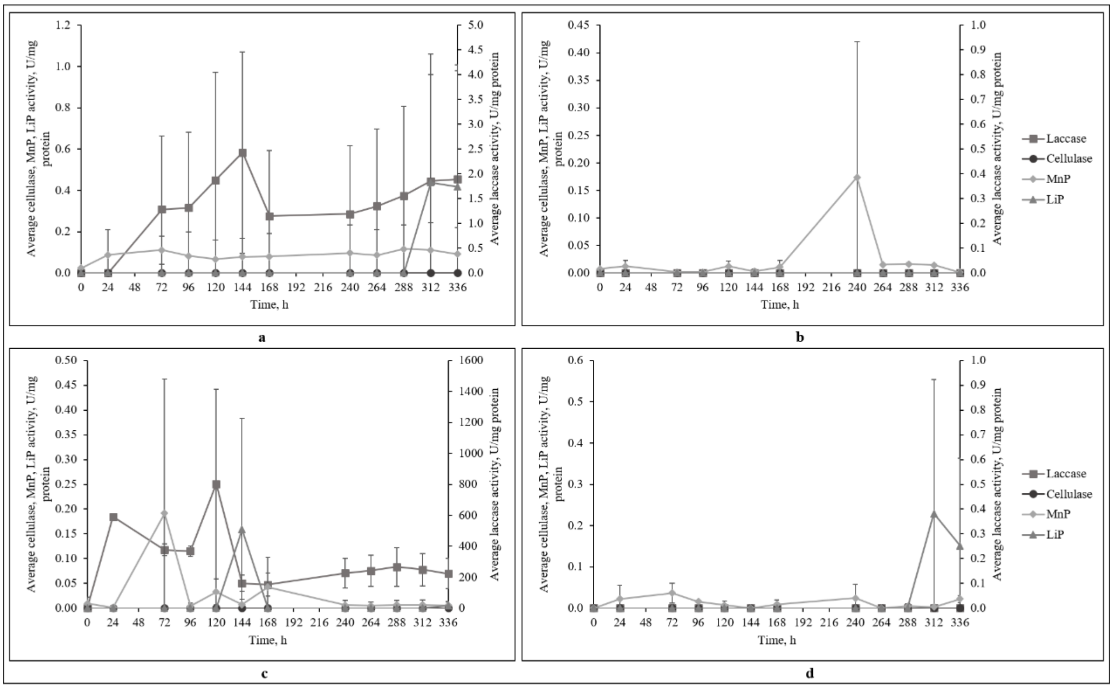

Figure S3. The activity of cellulase, manganese peroxidase (MnP), lignin peroxidase (LiP) and laccase (Units/mL) in (a) *I. lacteus*, (b) *B. adusta*, (c) *P. dryinus* and (d) *T. versicolor* cultures cultivated in the lignin – containing media.

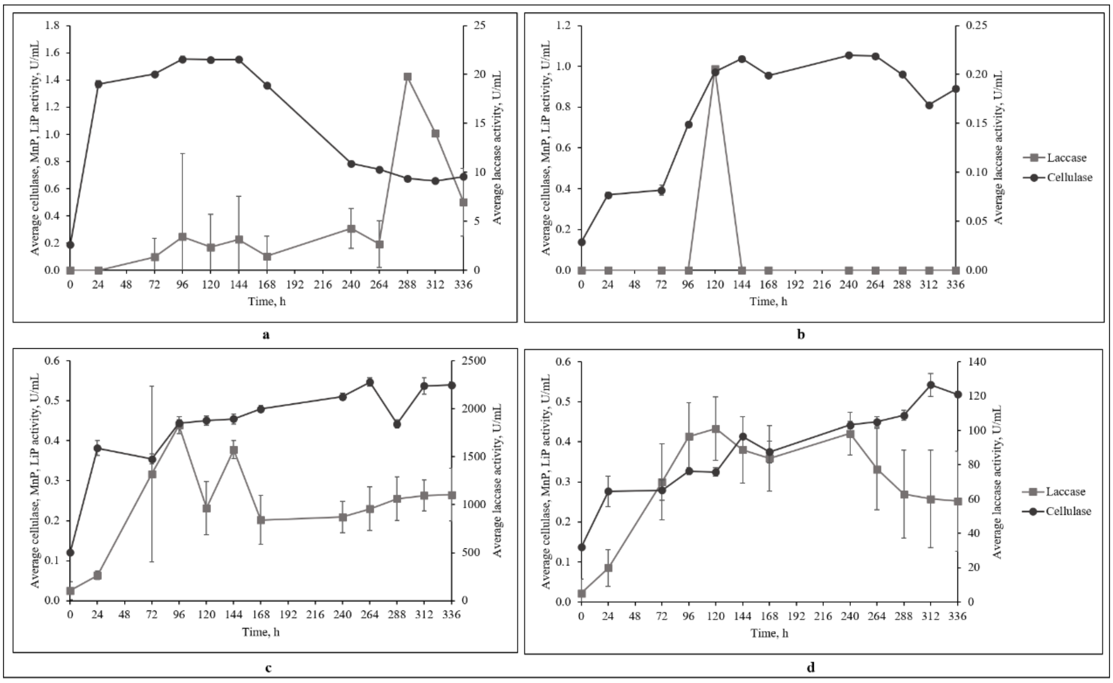

Figure S4. The activity of cellulase, manganese peroxidase (MnP), lignin peroxidase (LiP) and laccase (Units/mL) in (a) *I. lacteus*, (b) *B. adusta*, (c) *P. dryinus* and (d) *T. versicolor* cultures cultivated in the hay biomass – containing media.

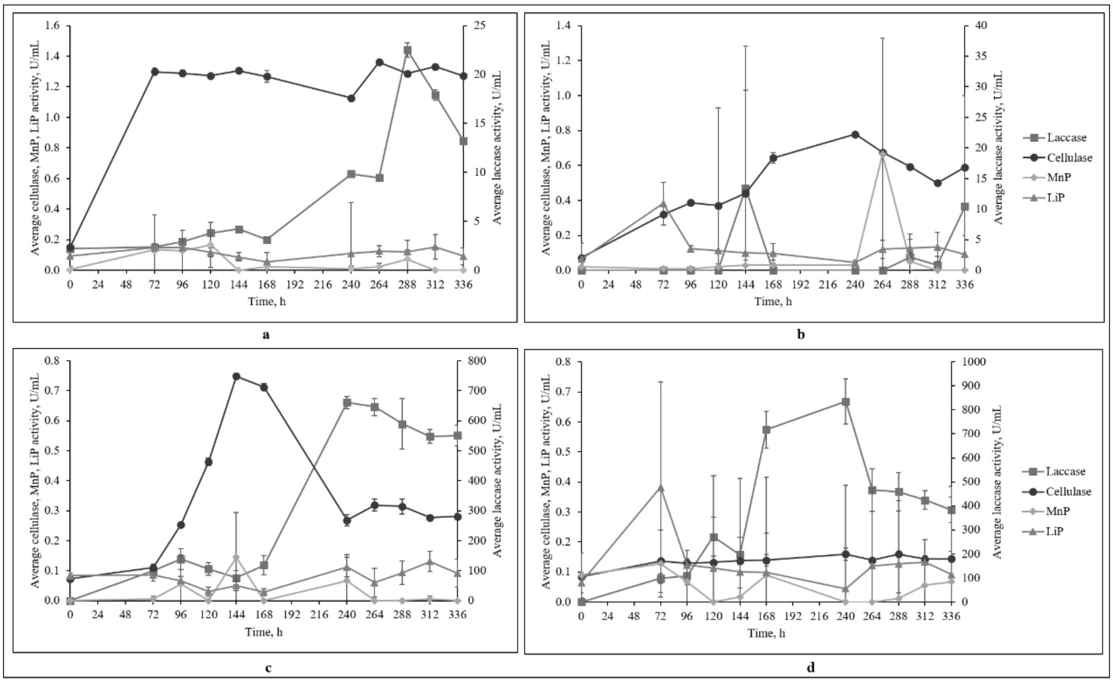

Figure S5. The activity of cellulase, manganese peroxidase (MnP), lignin peroxidase (LiP) and laccase (Units/mL) in (a) *I. lacteus*, (b) *B. adusta*, (c) *P. dryinus* and (d) *T. versicolor* cultures cultivated in the WWTP- derived screenings – containing media.

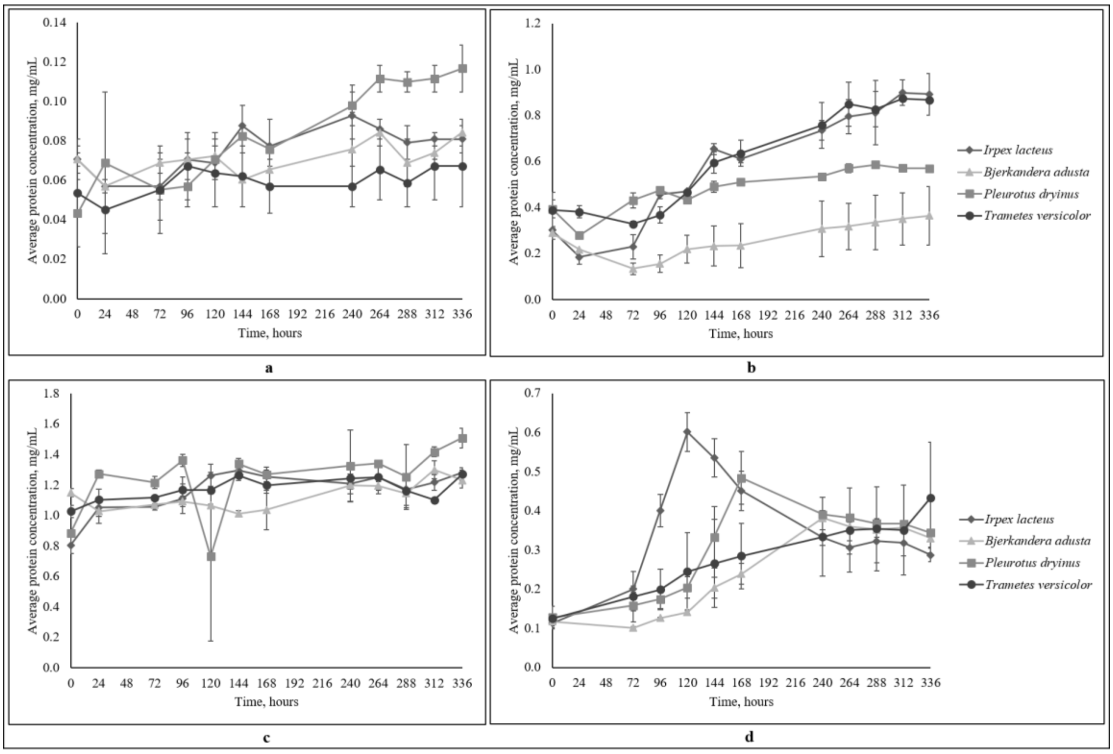

Figure S6. The concentration of total proteins in fungal cultures cultivated in the (a) glucose, (b) hay biomass, (c) lignin, and (d) WWTP- derived screenings – containing media.
